# Thinking in Pharmacy Practice: A Study of Community Pharmacists’ Clinical Reasoning in Medication Supply Using the Think-Aloud Method

**DOI:** 10.3390/pharmacy6010001

**Published:** 2017-12-31

**Authors:** Hayley Croft, Conor Gilligan, Rohan Rasiah, Tracy Levett-Jones, Jennifer Schneider

**Affiliations:** 1 School of Biomedical Sciences and Pharmacy, Faculty of Health and Medicine, The University of Newcastle, Callaghan, NSW 2308, Australia; Jennifer.Schneider@newcastle.edu.au; 2 School of Medicine and Public Health, The University of Newcastle, Callaghan, NSW 2308, Australia; Conor.gilligan@newcastle.edu.au; 3 Western Australian Centre Rural Health, Geraldton, WA 6530, Australia; Rohan.rasiah@uwa.edu.au; 4 Faculty of Health, University of Technology Sydney, Ultimo, NSW 2007, Australia; Tracy.Levett-Jones@uts.edu.au

**Keywords:** pharmacists, reasoning, medication supply, cognitive skills

## Abstract

Medication review and supply by pharmacists involves both cognitive and technical skills related to the safety and appropriateness of prescribed medicines. The cognitive ability of pharmacists to recall, synthesise and memorise information is a critical aspect of safe and optimal medicines use, yet few studies have investigated the clinical reasoning and decision-making processes pharmacists use when supplying prescribed medicines. The objective of this study was to examine the patterns and processes of pharmacists’ clinical reasoning and to identify the information sources used, when making decisions about the safety and appropriateness of prescribed medicines. Ten community pharmacists participated in a simulation in which they were required to review a prescription and make decisions about the safety and appropriateness of supplying the prescribed medicines to the patient, whilst at the same time thinking aloud about the tasks required. Following the simulation each pharmacist was asked a series of questions to prompt retrospective thinking aloud using video-stimulated recall. The simulated consultation and retrospective interview were recorded and transcribed for thematic analysis. All of the pharmacists made a safe and appropriate supply of two prescribed medicines to the simulated patient. Qualitative analysis identified seven core thinking processes used during the supply process: considering prescription in context, retrieving information, identifying medication-related issues, processing information, collaborative planning, decision making and reflection; and align closely with other health professionals. The insights from this study have implications for enhancing awareness of decision making processes in pharmacy practice and informing teaching and assessment approaches in medication supply.

## 1. Introduction

Dispensing is a core part of the medication management cycle and the role played by pharmacists [1]. The health system provides a safety mechanism by ensuring that pharmacists are responsible for providing an independent review of prescriptions before treatment commences, a critical check that remains separate from the prescribing process. In addition to the technical skills of labelling and supply of a medicine, dispensing also involves complex cognitive processes. The interpretation and evaluation of the prescription, including assessing the safety and appropriateness of the dosage, checking for contraindications and drug interactions, are examples of cognitive processes that occur during the process [1]. The ability to ensure that medicine dispensing is safe, accurate and appropriate requires a combination of thinking and decision-making, recognised as clinical reasoning. These skills have a profound impact on patient safety, yet this remains an unexplored area in the pharmacy domain [2]. 

The scope of pharmacy practice has extended beyond the supply of medicines in recent times, to include a growing range of patient-centred professional health services, however the traditional dispensing of medicines still remains an important priority for the majority of pharmacists. The expansion in the number and diversity of prescription medicines, including the exponential growth in complicated biological agents and generic drug brands, requires community pharmacists to determine the appropriateness of a wider range of medicines than ever before. There is a growing trend in pharmacies installing robotic dispensing systems to improve efficiency in medicine dispensing and while this approach removes some of the technical processes, supply of medicine still requires cognitive input by pharmacists to ensure the appropriateness of medicines for each patient and to deliver enhanced patient-centred consultations [3]. High level cognitive skills are required to decide whether the medication should be handed to the patient cannot currently be undertaken by automated dispensing systems [4]. Furthermore, integral to new and expanding roles for pharmacists, which include new responsibilities such as extending or modifying prescriptions is the responsibility for making clinical decisions, both independently and collaboratively [5].

Clinical reasoning is a complex process that depends on the ability of humans to process, memorise, recall and synthesise huge amounts of data. These are all vulnerable areas that ultimately impact on healthcare professionals’ competency and clinical performance [6]. For pharmacists, the process of reviewing a prescription or a medication chart and responding to patient symptoms are processes that are considered to be logical and systematic. However, clinical reasoning is complex and includes many overlapping and parallel processes [7,8]. The ability for a health professional to provide safe, high-quality care can be dependent on their ability to reason, think and judge. The World Health Organisation (WHO) stipulate that decision-making is a critical component of workplace safety in relation to minimising errors [9]. A majority of research in decision-making relates to doctor’s diagnosis or treatment decisions [10], however there is increased published literature for factors affecting the pharmacists performance, including decision making and the incidence of dispensing errors [2,11,12,13]. The need for scientific evaluation of decision-making processes has become increasingly apparent in order to address the unexplained variability in performance, high rates of medication error and increased health expenditure. As a result, increased attention is being directed towards the development of valid and reliable methods to assess healthcare professionals’ decision making and clinical reasoning skills [14]. 

Clinical reasoning is a core competency for all healthcare professions but it is not always clear how the reasoning processes used in each profession differ from each another. Current knowledge of pharmacists’ clinical decision-making largely draws on studies undertaken in other health disciplines [8,15]. Several theories exist in the literature that relate to the reasoning processes that clinicians use throughout a consultation [15]. The information-processing/hypothetico-deductive approach to cognitive reasoning has a long history in medical education and practice [16]. This approach involves several stages including cue recognition (collate clinical patient information), hypothesis generation (tentative explanation based on initial information), cue interpretation (focus on information from a number of sources) and hypothesis evaluation (collate and evaluate evidence that supports or rejects the original hypothesis). By comparison, the intuitive-humanist model focuses on intuition and takes into account the impact of clinical experience on decision-making processes [15]. The evolution of these theories suggests that clinicians may use a combination of intuition and analysis in their consultations [15]. More recently, clinical reasoning models have been used to describe the complex process by which nurses collect cues, process information, understand the patients’ problem, implement interventions, evaluate outcomes and reflect on the process [17].

The clinical consultation is the practical embodiment of the clinical reasoning process [15]. Various models of consulting have been identified in the literature, however they rarely focus on medication-related issues and are not ideal for evaluating medication-specific consultations. One exception to this is the Medication Related Consultation Framework (MRCF), a validated tool developed specifically for teaching and evaluating patient-centred, medication-related consultation skills by pharmacists [18]. However, whilst it provides a framework for the consultation process, the MRCF does not take into account the specific decision-making processes pharmacists use when reviewing a prescription and deciding if the prescribed medicine may be safely supplied to the patient, such as establishing if the medication order meets legal requirements; verifying the appropriateness of the drug, brand, form, strength, quantity; or decisions that relate to selection and assembly of a dispensed medicine. 

Through key stakeholder consultation the pharmacy profession has already developed guidelines that inform the key steps that a pharmacist should follow when supplying a prescription [1]. There are similar guidelines in other jurisdictions [19] as well as internationally accepted standards for dispensing practice [20]. However, this framework does not detail the complex reasoning processes used by pharmacists to inform each of the steps. Compared to other professions, there is a lack of understanding and knowledge of the many processes used by pharmacists as they unravel the myriad of cues and leads associated with determining the appropriateness of a medicine order for a given patient [21].

The think-aloud method has been previously used to for providing insights into pharmacists’ decision making patterns and has functioned effectively in this context [5,7]. In 2015, community pharmacists in the United Kingdom (UK) setting were asked to think-aloud their thoughts while establishing the cause of a simulated patient’s symptoms. This study effectively highlighted that although most pharmacists arrived at the right diagnosis, the ability to clinically reason were limited. More recently, in a preliminary exploratory study in Canada, community pharmacists were asked to verbally reason through their decision-making process when presented with a paper-based case study dealing with challenging situations. This study too was able to highlight opportunities for educators to consider new ways of preparing pharmacists [5]. The aim of this study was to explore the reasoning processes community pharmacists undertake when presented with a prescription in a simulated patient scenario. 

## 2. Materials and Methods

A qualitative, descriptive study was used to analyse cues associated with the decision-making processes pharmacists used while reviewing and supplying prescribed medicine in a simulated patient-pharmacist encounter. This methodology has previously been used to examine the clinical reasoning process used by other healthcare professionals [22,23,24]. 

Each pharmacist was asked to verbalise their thoughts spontaneously while performing tasks in patient management (concurrent think aloud using short term memory (STM)), which incorporates narration on medicines related aspects of patient care during the patient counselling component to facilitate clinical reasoning. This initial simulated patient-pharmacist encounter was video/audio-recorded. Each participant also then completed a post-task interview with the researcher to further investigate the rationale for specific actions or to elaborate on comments made during the simulation. During the follow up interview the video was played back to the pharmacist with an iPad to enable video-stimulated recall, in addition to a series of semi-structured interview questions, as prompts for thinking aloud (Appendix A) (retrospective think aloud using long-term memory (LTM)) [25,26,27]. The post-task interview was also audio-recorded to facilitate data analysis. 

A scenario was developed (Box 1) between two pharmacy academics and a simulation expert, with objectives that closely align with the professional competency standards in Australia for medication supply. The scenario was further developed iteratively following input from two practicing community pharmacists, prior to being used in the think aloud study. The simulation scenario represents a typical patient-pharmacist encounter, with decision-making focused on a request for a prescription-only medication supply to a female type 2 diabetic patient. The simulation was conducted in a simulated community pharmacy at the University of Newcastle, Australia (UON). The scenario involves a range of concepts that have a direct or indirect impact on the safe management of diabetes including monitoring of blood glucose levels, assessment of renal function, responding to patient signs and symptoms, management of medication related issues and information required for patient counselling. The study was limited to one clinical scenario because the aim of the study is to explore the core cognitive processes used to make decisions about prescribed medicines, which should apply to any given scenario.

Box 1 **Simulation scenario in prose.**

A 60-year-old female patient presents to a community pharmacy to collect insulin glargine (10 units nocte, Qty 5) and amoxycillin + clavulanic acid (875/125mg BD, Qty 10) on a prescription written by her regular GP. These prescriptions have been handed to a pharmacy dispensary technician who has processed the prescriptions using FRED dispense and generated labels for each item in preparation for the pharmacist to complete the dispensing procedure. Pharmacist participants are to assume they are in their community pharmacy and their role is to manage this patient who would like to pick up her prescriptions. As the pharmacist manages the patient, they ‘think aloud’ about what they are doing by verbalising their thoughts and behaviours.

Ten Australian registered community pharmacists who are currently practicing in a community pharmacy were recruited using a mixture of non-probability sampling methods - convenience and snowball sampling. Each pharmacist individually participated in a simulation and evaluation for saturation of the data was conducted after each observation. [27]. The competencies embedded in the reasoning task are those that are expected of all entry level graduates and therefore no additional training was required for pharmacists in order to engage in the scenario, however, prior to data collection, participants were given instructions and a briefing about the think aloud technique [27].

The deductive approach used for the directed content analysis provided initial coding categories informed by a preliminary analysis of clinical decision-making literature. Two existing clinical reasoning frameworks for health professionals: the *clinical reasoning cycle* for nursing practice and the *biopsychosocial model of clinical reasoning* underpinning physiotherapist’s assessment and management of a patient, were identified as existing frameworks that would provide an initial approach to organising the research data. Through observation the data showed that the pharmacist’s decision-making processes correlated closely with the categories derived from the existing clinical reasoning cycle in nursing practice. The initial coding categories for clinical reasoning (consider patient situation; collect cues/information; process information; identify medication-related issues; set goals; take action; and evaluate outcomes) were applied to the data to conduct a preliminary analysis [8,17,18,27,28]. The data was then mapped to the specific component of the clinical reasoning cycle, which included a general description of the pharmacists cognitive process and evidence using specific dialogue examples from concurrent and retrospective think aloud data. 

The codes were further developed iteratively and adapted to the pharmacy context by drawing on key themes from the biopsychosocial model of clinical reasoning used in physiotherapy and the Pharmacy Guild of Australia Medicine Dispensing Process [1]. The initial analysis of the decision- making process was based on the concurrent think aloud data for all participants. Once this was complete, the retrospective think aloud data was analysed to fill gaps and provide further explication. Ethical approval was granted from the Human Research Ethics Committee, University of Newcastle.

## 3. Results

Ten pharmacists participated in the think aloud study and Table 1 summarises the characteristics of the participant group. The results are presented in two sections. Firstly, how the pharmacists’ thought processes correlated with existing medicine dispensing processes and secondly, key themes in clinical reasoning identified for pharmacists to make a decision.

### 3.1. Pharmacist Performance

All of the pharmacists demonstrated distinct patterns in verbalisation about the required tasks and each of them dispensed the medicines in accordance with the *Medicine Dispensing Process* model [1]. There were two distinct patterns in the order in which actions were performed. Seven pharmacists initially performed a check of the prescription and the dispensed product and then engaged the patient to further clarify patient specific details. Three pharmacists engaged the patient immediately after accepting the prescription to gather information, prior to performing a final check of the dispensed medicine. There were no differences in expected outcome for the patient irrespective of the process used by the pharmacist (see Table 2).

### 3.2. Key Themes in Clinical Reasoning in Describing How Pharmacists Arrived at Their Decision

Here, the results are presented using descriptors that relate to the clinical reasoning framework developed for nursing practice [17] and adapted for the reasoning task as it relates to a community pharmacist performing medication supply. The descriptors include: review of prescribed medicine order, retrieving information, processing information, identifying medication-related issues, collaborative planning, decision making and reflection. 

#### 3.2.1. Review of Prescribed Medicine Order

All pharmacists initially reviewed the prescription to understand and interpret the information and then place it in context by establishing a sense of what the patient situation was. Pharmacists at this point were initially concerned with the issues outlined in Table 3 which are common to the review process: 

#### 3.2.2. Retrieving Information 

This is defined as the process of collecting cues from a source of information to use as a foundation for planning patient-orientated decisions relating to medicine management. Pharmacists collected information from a number of sources, including the patient, dispensing software and drug information resources, as well as drawing on their own discipline-specific knowledge, as outlined in Table 4. This thinking process involves noticing *(e.g., pharmacist 3, ‘I noticed in your dispensing history that you are already using an injection’);* and reflecting *(e.g., pharmacist 6, ‘it is quite a broad-spectrum antibiotic and I have seen it used for a number of different infectious conditions’)*. 

#### 3.2.3. Process Information

This is defined as the process of interpreting and clarifying information. This was mainly observed when the pharmacists compared more than one piece of information to another and drew comparisons in order to further organise information *(e.g., pharmacist 8 ‘the patient has an antibiotic prescribed and they are diabetic so I know if they are acutely unwell this can affect their blood glucose levels and the subsequent need for insulin’)*. Table 6 outlines the cognitive processes that pharmacists used in interpreting information. Seven of the pharmacists verbalised that when collecting cues and information provided on the prescription they were looking for information that stood out as unusual or different compared to what they were used to seeing in their practice role *(e.g. pharmacist 10 ‘so when I am looking at the prescription I am checking to see if all the medicine details are consistent with standard dosing guidelines and what you would normally see in practice’)*. 

#### 3.2.4. Identification of Medication Related Issues

During the dispensing process pharmacists recognised key issues that were directly relevant to their role in meeting the pharmaceutical care needs of the patient. These issues have been broadly classified into two groups: (1) medication-related issues that relate to the presenting prescription which need to be addressed for the patient to achieve optimal benefits from their medicines with minimal risk of adverse events; and (2) co-existing issues which do not directly affect the decision about the supply of the prescribed medicine. There were four immediate issues and two co-existing issues that were common to all pharmacists in the simulation outlined in Table 5.

#### 3.2.5. Collaborative Planning

All pharmacists engaged in mutual decision making with the patient during the process of considering various choices of actions and explaining the options available. Pharmacists actively sought the patient’s opinions and relied on this information to inform their decision making. This process incorporated two main cognitive processes; eliciting the ideas and opinions of the patient and anticipating what to expect. Table 6 provides examples of the cognitive processes that pharmacists used in collaborative planning.

#### 3.2.6. Decision Making

All 10 pharmacists met the patient’s pharmaceutical needs and correctly arrived at the final decision that the prescribed medicines were safe and appropriate to supply to the patient. All pharmacists decided to provide pharmacological management, by recommending that the patient be supplied with both prescribed medicines and they provided verbal counselling and written information. The pharmacists also decided the patient would require specific follow up on the medications that were being supplied, including review of diabetic leg ulcer in 5 days’ time, more frequent ambulatory blood glucose monitoring and referral to diabetes educator. They also confirmed that gliclazide should be ceased to minimise the additive risks of hypoglycaemia when starting insulin. The main cognitive process used was rationalisation, defined as the process of justifying the thoughts and actions. Table 6 provides examples of the cognitive processes that pharmacists used in decision making. 

#### 3.2.7. Reflection

Each of the pharmacists showed metacognitive skills, an awareness of their own thinking, during both concurrent and retrospective think aloud processes. They reflected on their management of the patient, agreed that this was a common scenario that they were likely to face in their usual practice role and contemplated what this situation may have been like if encountered in real practice *(e.g., pharmacist 2 ‘if it were a real situation and I took the time I needed to ensure the patient was managed appropriately, I’d probably have about 10 more prescriptions from other consumers waiting for me to check, so in reality sometimes there is an inability to provide a complete consultation with patients, especially if you are the only pharmacist on duty’)*. The pharmacists also noted that prioritising their time was one of the key influences on how they managed the medication supply *(e.g., pharmacist 9 ‘it is important to make judgement of your own time pressures and those of the patient about whether they are in a hurry to catch a bus or need to go to work or whether they are happy to keep talking because when they have a number of health conditions and chronic health conditions it is about prioritising what information you are able to give them in the short time you have available’)*. 

### 3.3. Other Observations

Pharmacists with more than 20 years’ experience were on average faster at arriving at their decision that supply of the medications to the patient was appropriate. The average time for pharmacists with more than 20 years’ experience was 6 min and the average time for pharmacists with less than 20 years’ experience was 12 min. However, many other factors may have influenced their dispensing efficiency such as recency of practice, current work load, usual practice model etc. There were no differences noted between male and female pharmacists. 

## 4. Discussion

Our findings identified seven different processes that were sequentially performed by pharmacists to ensure the pharmaceutical needs of the patient were met when presented with a written prescription. The results show that all pharmacists essentially apply the steps recommended in the Medicine Dispensing Process model and arrived at the decision that the prescribed medicines were safe and appropriate to supply to the patient. However, the focus of this study was to unravel the processes involved in a pharmacist arriving at this decision. Using a community pharmacy simulation, the pharmacists progressed from one form of thinking to another, moving back and forth among various thinking processes, in arriving at a decision on the safety, accuracy and appropriateness for dispensing a medication. 

The seven reasoning processes are drawn from existing frameworks and modified to suit the medication supply context in a community pharmacy. The key themes aligned well with other models of clinical reasoning and demonstrated similarities between the way pharmacists and other health professionals think. The clinical reasoning process developed from a body of research undertaken by [17,29] was used as a basis for categorising the reasoning processes shown by pharmacists in the think aloud study, with a high degree of correlation. Like physiotherapists and nurses, a pharmacists’ reasoning begins with the initial collection of cues and information, which forms the basis of the working interpretations as the reasoning process continues. For instance, when pharmacists initially reviewed the prescription, the initial interpretations include hypothesis about what condition the patient is likely requiring medication for; and then this information is considered against subsequent information that is obtained throughout the consultation that supports or refutes the initial impressions. 

Further similarities with physiotherapists and nurses were the element of routine that pharmacists demonstrated in their decision-making process and the categories of information they used in problem identification and arriving at their decision. Through professional practice it was obvious that pharmacists used common sources of information that was useful for identifying medication-related issues and developing management strategies. Although this study looked only at one specific simulated encounter, it is envisaged that beyond this example, pharmacists’ reasoning processed would include specific enquiries related to the patient’s individual situation. 

The pharmacists were engaged in dealing simultaneously with multiple tasks and problems when presented with the prescription. For example, they verbalised that they had to think about the validity and legality of the prescription, the therapeutic aspects of the medication order and integrating this with existing knowledge and patient information. What was observed is that pharmacists tended to engage in thinking processes that were involved in a specific task so that the focus of their attention was on one component of the dispensing process, before moving onto the next. The pharmacists varied in terms of where they decided to focus their attention. Some spent more time reviewing the medication order and focused their attention on clarifying the intentions of the prescriber to determine which formulation of insulin they would dispense. Others focused their attention on consulting with the patient in a process of collaborative planning about how the patient would manage the administration of their new medication. 

When the pharmacists were required to consider multiple pieces of information at one time, they showed distinct patterns of cognitive processing in merging the information together and to identifying specific medication related issues. One of the main issues for the simulated patient was their increased risk of hypoglycaemia when supplying insulin in combination with existing medications. To recognise this potential clinical problem, the pharmacists demonstrated predictive reasoning (anticipating an outcome based on existing therapeutic knowledge and/or experience); and forward reasoning (obtaining new information from the prescription, the patient and the dispensing history to substantiate a hunch) to determine the potential issue. 

The pharmacists used a variety of information sources as they progressed through the reasoning process. A thorough review of information provided on the prescription and in the patient’s dispensing history were key in identifying discrepancies or alterations in drug, strength, dose, dosing frequency, quantity, drug formulation and drug interactions. All of the pharmacists agreed that they would be unable to determine the appropriateness of a medication order without obtaining information about the medical diagnosis but interestingly, this information is not a mandatory requirement for medication orders and is therefore not usually available to a pharmacist without consulting the patient. There is a risk with making assumptions about the indication for use of a prescribed medicine, as many drugs have multiple potential indications and specific doses and duration of use associated with each. Furthermore, an increasing number of medicines are being used beyond the scope for which they are usually recognised for use. This provides a challenge for pharmacists who are then required to elicit such information through careful medication history taking. Similarly, pathology information, such as fasting BGL and HbA1c data relevant to the simulation in this study, are examples of information not routinely available to pharmacists reviewing a medication order, yet these were identified as important for deciding whether a particular medication dose would be appropriate. 

All of the pharmacists decided that the patient in the simulation would benefit from referral to other healthcare professionals (HCPs), for example a diabetes educator. While pharmacists are able to make recommendations to the patient that they pursue follow up with other HCPs, within the Australian context there are no pathways for pharmacists to formally refer to another member of the multidisciplinary team.

Additional dimensions of clinical reasoning were identified from the think aloud data which could be considered to be limitations to the study. The pharmacists reported that their decision making could have been impacted by their interaction within the unfamiliar community pharmacy environment where the simulation to place (contextual interaction). Medicine dispensing in community pharmacies relies on a number of sequential steps with familiar task orientations for the pharmacist including position of stationery, position of medications on the shelf and usual drug information resources. 

Further, the spontaneous verbalisation of thoughts (concurrent think aloud) while performing the dispensing task could have disrupted the pharmacists’ train of thought and therefore may have altered their decision-making process. Two pharmacists suggested that the think aloud process was disruptive but that it was unlikely to have altered the way they managed the patient. However, other pharmacists found the think aloud process to be quite natural and even beneficial, with one citing they often ‘talk aloud’ as an instinctive process during some medicine dispensing tasks in practice. The main influence on the reasoning process described by pharmacists was timing, in that pharmacists described usually having less time to deal with the type of task presented in the simulation. Further limitations to the study include the focus on only one simulation encounter. Thus, future work in this area could investigate decision making processes using a wider range of topics and medications. 

Although the simulation scenario, simulated patient and demonstration pharmacy provide an authentic representation of a typical patient-pharmacist encounter, there were some aspects of the community pharmacy environment that were not captured during the simulation. For example, in the same consultation in a real-life pharmacy situation pharmacists are commonly engaged in multitasking activities for more than one patient and may also be exposed to a variety of disturbances such as noise and interruptions from other staff and consumers. In the simulation, the pharmacist’s time was allocated purely to the specific intervention and there was less need to consider some aspects of the consultation such as privacy. 

One of the key influences on the overall reasoning process described by pharmacists was generally the limited time to complete dispensing tasks. In the simulation, pharmacists were not subject to all the usual time pressures of a typical community pharmacy, for example, multiple prescriptions to handle, or interruptions such as assisting another consumer with a higher priority. However, because pharmacists recognised they would usually have limited time to deal with the type of task presented in the simulation, they followed the same principles of time management during the simulation, in the priorities they placed on their actions and decisions relating to how they chose to manage the overall consultation.

Because this is a preliminary exploratory study the findings cannot be extrapolated too broadly, however this is an important step in better understanding decision-making for a profession that continues to investigate what factors lead to medication errors in community pharmacy. The actions demonstrated by pharmacists in this study provide information about how pharmacists approach decision–making in medicine dispensing and provides opportunities for educators to use this to enhance teaching and evaluating competency. 

## 5. Conclusions

Pharmacists are required to make decisions about the safety and appropriateness of prescription medications for large numbers of patients with diverse health needs. The reasoning skills used by pharmacists impact on patient safety and can have adverse effects on patient outcomes. All of the pharmacists in this study correctly supplied the prescription medication in the community pharmacy simulation, however, the think aloud technique uncovered a complexity of reasoning processes that led to this outcome. It is not surprising that the reasoning processes used by pharmacists align with those of other health science professions and can be defined within seven core dimensions of reasoning as relating to the review and supply of prescribed medicines. Understanding and promoting an awareness of the systematic and complex process that guide decision-making by pharmacists, could contribute to enhancement of these clinical reasoning processes. Furthermore, these findings could inform the development of a robust model to guide educational interventions to improve the training of pharmacists and the care they provide to patients.

## Figures and Tables

**Table 1:** Demographic data for pharmacist participants.

Demographic		Number of Pharmacists
Gender	Male	3
	Female	7
Pharmacy experience	<2 years	1
	2–10 years	6
	11–20 years	1
	>20 years	2

**Table 2:** Order of processes/actions taken by each pharmacist participant.

Step in Medicine Dispensing Process	Pharmacist Participant									
	1	2	3	4	5	6	7	8	9	10
1. Check prescription details	1	1	1	1	1	1	1	1	1	1
2. Script validity	3	2	3	6	5	7	2	2	2	2
3. Safety and appropriateness	4	4	5	7	4	6	5	4	4	5
4. Review dispensing history	2	3	4	3	3	4	3	3	3	3
5. Patient specific factors	5	6	2	2	2	5	4	5	5	4
6. Select product/check selected product	6	5	6	4	6	2	6	6	6	6
7. Dispensing check ^a^	7	7	7	5	7	3	7	7	7	7
8. Supply prescription to patient/carer: re-check	8	8	8	8	8	8	8	8	8	8
9. Counsel patient on safe and appropriate use	9	9	9	9	9	9	9	9	9	9
Expected Outcome For Patient ^b^	Yes	Yes	Yes	Yes	Yes	Yes	Yes	Yes	Yes	Yes

^a^ Step 8 in the medicine dispensing process “label and assemble dispensed products” was completed by a dispensary assistant prior to pharmacists undertaking their checking procedure; ^b^ Defined as to whether the pharmaceutical needs of the patient have been met.

**Table 3:** Immediate review by pharmacist.

Issue Category	Common thought Processes	Example from Data
Nature of Medication	Does the drug have a narrow therapeutic window? Is this a new medication with limited experience, or one that I have not dispensed before? Will this medication require additional/specific counselling requirements such as device demonstration?	*‘Amoxycillin is a common medication with a wide margin of safety’* *‘The patient has not used insulin before and there is lots of information I will need to go through including demonstrating the injection’*
Patient	Is this for an adult or a child? Is this patient acutely unwell? Do I know the patient—am I likely to have a good dispensing history? Is the patient in a hurry?	*‘The prescription appears to be for an adult—they are a local patient so may have been to this pharmacy before’*
Prescription	Is the prescription legal? Is there any information missing? Does the medication attract financial subsidy or is it expensive for the patient?	*‘My first concern is if the prescription is legitimate and legal’* *‘I usually glance at the prescription to see if there is anything that stands out as unusual or if there is missing information’* *‘I always check if it is a medication that attracts a government subsidy because then there will be extra details that I need to check such as concession details’*

**Table 4:** Sources of information pharmacists retrieved and used in reasoning.

1. Dispensing history	√	√	√	√	√	√	√	√	√	√
2. Prescription-legalities	√	√	√	√	√	√	√	√	√	√
3. Patient–medication history	√	√	√	√	√	√	√	√	√	√
4. Patient–medical history	√	√	√	√	√	√	√	√	√	√
5. Patient–pathology/diagnostic data	√	√		√	√	√	√	√		
6. Patient–preferences	√	√	√	√	√	√		√	√	√
7. Patient–other e.g., financial entitlements, compliance	√	√	√	√			√			
8. Propositional knowledge derived from theory	√	√	√	√	√	√	√	√	√	√
9. Non-propositionalknowledgederivedfromprofessional/personalexperience	√	√	√	√	√	√	√	√	√	√
10. Drug information sources–evidence-based guidelines	√						√	√		
11. Drug information sources–product information	√	√								

**Table 5:** Immediate issues and co-existing issues identified and action(s) taken by pharmacists.

**IMMEDIATE ISSUES IDENTIFIED**	**ACTION(S) TAKEN BY PHARMACISTS**
1. Recent unstable glycaemic control (pathology, patient) and the need for changed/additional pharmacological intervention	Determine the rationale for the prescribed medication (insulin) and check appropriateness before supply
2. Current infection (venous leg ulcer) and the need for pharmacological intervention	Determine the rationale for the prescribed medication (antibiotic) and check appropriateness before supply
3. Drug-related precaution—duplication of hypoglycaemic agents predisposes to increased risk of hypoglycaemia	Clarify with patient changes to existing medicines that include cessation of gliclazide and exenatide with continued metformin use
4. The patient is commencing on new medicines requiring explanation of any changes/recommendations/device demonstration	Provide medicines information for patient including administration, dose, insulin injection technique
**Co-existing Issues Identified**	**Action(s) Taken by Pharmacists**
1. Patient is complacent towards non-pharmacological management of diabetes	Provide lifestyle advice to aid management, offer some education as to the importance of good self-management of diabetes
2. Patient has not been referred to diabetes educator and has not seen a diabetes specialist for a couple of years	Recommend referral to diabetes educator

**Table 6:** The phases of clinical reasoning process ^1^ with descriptions for the pharmacy context and examples from the think aloud data.

Process	Description	Example of Pharmacists’ Thinking
**1. Consider prescription in context**	**Review** legal and therapeutic aspects of prescribed medicine order **Describe** patient and context	*I can see that an adult female patient is collecting a prescription for a penicillin antibiotic and insulin. I’m just checking the script to see if it is legal and valid—if it’s in date and the medication order is signed by the prescriber.*
**2. Retrieving information**	**Gather** medication history from patient	*Do you have any allergies, particularly to penicillin?*
	**Review** dispensing history, laboratory/diagnostic information	*“I would establish if these are new medicines for this patient or if they have changed by looking up their dispensing history.”* *“I am asking about BSL levels to ascertain the level of diabetes control and look at medication administration in the context of overall disease management.”*
	**Recall** information from past/previous experience	*“I have seen diabetic patients with infections have fluctuating and higher than usual blood glucose levels.”*
	**Investigate** new information e.g., directed searching in drug information databases	*I am just going to check the therapeutic guidelines to see if this is the right duration of antibiotic treatment for a diabetic leg ulcer.*
**3. Processing information**	**Recognise** the difference between normal and abnormal by comparing information	*What I’m looking for is if there is anything unusual or different about this prescription that stands out compared to what I am used to seeing.*
	**Distinguish** between information which is relevant from irrelevant;	*The antibiotic prescribed is penicillin, so I need to be looking for allergies but Matilda has no history of penicillin allergy. For this script we don’t need be concerned about her morphine allergy.*
	**Relate** information to identify patterns of information	*I see that oral hypoglycaemic agents have not achieved optimal [diabetes] control and lifestyle interventions have not helped BSL levels and now there are some complications of high blood sugar starting to appear, including leg ulcer. So Matilda’s diabetes control is deteriorating.*
	**Match** similar information and/or: identifying a **mismatch** between two pieces of information	*Matilda has been using the Byetta and that requires injections, so this information tells me how acceptable administering a new drug [insulin] in the same form would be.* *So that immediately makes me pull up as to why a doctor would be prescribing an antifungal for an ulcer. I can’t think of any kind of therapeutic reason why that would be the case so that would require further investigation.*
	**Prioritise** information by ranking its importance	*Matilda has a number of chronic health conditions, so it is about prioritising what information you are able to give her in the short time you have available.*
**4. Identifying medication-related issues**	**Synthesise** information to formulate immediate issues that need to be addressed	*There is a duplication of hypoglycaemic agents that makes hypoglycaemia more likely in this patient.*
	Secondary issues that need to be addressed	*I can see the patient is complacent about their lifestyle aspects of diabetes management.*
**5. Collaborative planning**	**Elicit** ideas and opinions	*Tell me how you feel about starting insulin and going home tonight to administer for the first time.*
	**Anticipate** what to expect	*The antibiotic is broad spectrum and may cause diarrhoea or thrush. I could recommend a probiotic to minimise the chance of this occurring and am asking how Matilda would feel about this, because it will be an extra expense and extra medication to take.*
**6. Decision making**	**Verify** correct information	*I look at the drug information on the script and check that against the dispensed item. I am checking the name of the medication [amoxycillin + clavulanic acid] and its strength [875/125] against both the label on the product and the box itself. Then the directions [1 tablet every 12 h]. Then I check the quantity [10] so this is all correct.*
	**Justify** thoughts and actions	*Insulin and metformin is an acceptable combination for Type 2 diabetes and the prescription is entirely legitimate.*
	**Select** appropriate interventions to optimise patient outcomes	*I recommend Matilda go back to her GP and the GP will measure the outcomes of the new medications. A diabetes educator can assist with overall disease state management. She could also come back to the pharmacy, to get her blood glucose measured, have a HMR or diabetes MedsCheck, have their BP monitored.*
**7. Reflection**	**Contemplate** what was done well and what could have been done differently	*I should have asked more about their reflux—it could have been related to diabetic gastroparesis.* *I would not usually have this long to spend with a patient in the pharmacy.*

^1^ Adapted from [17,29,30,31].

## Appendix A. Semi-Structured Follow-Up Interview with Example Prompts.

Initial participant debriefing
  1) Discuss how you are feeling after yoursimulation experience
  2) Summarise the task and the outcomes of your actions. What did you do and what were the outcomes for the patient?
Data collection using video cues
  3) Talk me through your thoughts after you were handed the prescription from the patient
  4) What came to mind when you were undertaking the initial assessment of the prescription?
  5) Discuss the process you went through to dispense the medication for the patient
  6) *Include questions that further investigate specific actions of behaviours of the participant that were not raised spontaneously during the post-task discussion, e.g., why did you look up that information? What was the rationale for asking that question? How did you arrive at that conclusion?
Data collection based on reflections
  7) Discuss any challenges that you identified/needed to overcome in supplying this medication to the patient
  8) Discuss the information sources used to consider the appropriateness of the prescribed medicine
Conclusions
  9) Do you have any further information that you feel would assist with our understanding of the decision-making process that are required when supplying medications?
Are there any further comments you would like to make about the simulation task?
